# A QUICK NOVEL MOBILE HEARING IMPAIRMENT ASSESSMENT FOR DIGITAL SPEECH HEARING SCREENING

**DOI:** 10.1093/geroni/igae098.3329

**Published:** 2024-12-31

**Authors:** Russell Banks, Barry Greene, Isaiah Morrow, Marissa Ciesla, David Woolever, Sean Tobyne, Ali Jannati, Alvaro Pascual-Leone

**Affiliations:** Linus Health, Boston, Massachusetts, United States; Fire1, Glasnevin, Dublin, Ireland; Linus Health, Boston, Massachusetts, United States; Linus Health, Boston, Massachusetts, United States; Lucid Hearing, Wake Forest, North Carolina, United States; Linus Health, Boston, Massachusetts, United States; Linus Health, Boston, Massachusetts, United States; Hebrew SeniorLife, Boston, Massachusetts, United States

## Abstract

It is estimated that 1 in 4 people worldwide will be living with hearing impairment by 2050. We propose a digital Speech Hearing Screener (dSHS) using short nonsense word recognition to measure speech-hearing ability. We compare dSHS outcomes with standardized pure-tone averages (PTA) and speech-recognition thresholds (SRT). 50 participants aged 55 or older underwent PTA and SRT measurement. One-way ANOVA was used to compare differences between hearing impaired and hearing not-impaired groups, by the dSHS, with a clinical threshold of moderately impaired hearing at 35dB and severe hearing impairment at 50 dB. dSHS results significantly correlated with PTAs/SRTs. ANOVA results revealed the dSHS was significantly different (F(1,47)=38.1, p< 0.001) between hearing impaired and unimpaired groups. Classification analysis using a 35dB threshold, yielded accuracy of 85.7% for PTA-based impairment and 81.6% for SR-based impairment. At a 50dB threshold, dSHS classification accuracy was 79.6% for PTA-based impairment (NPV-93%) and 83.7% (NPV-100%) for SRT-based impairment. The dSHS successfully differentiates between hearing-impaired and unimpaired individuals in under 3 minutes. This hearing screener offers a time-saving, in-clinic hearing screening to streamline the triage of those with likely hearing impairment to the appropriate follow-up assessment, thereby improving the quality of services. Additionally, this tool can help to rule out hearing impairment as a cause or confounder of cognitive impairment.

